# Zinc and Copper Ions Differentially Regulate Prion-Like Phase Separation Dynamics of Pan-Virus Nucleocapsid Biomolecular Condensates

**DOI:** 10.3390/v12101179

**Published:** 2020-10-18

**Authors:** Anne Monette, Andrew J. Mouland

**Affiliations:** 1 Lady Davis Institute at the Jewish General Hospital, Montréal, QC H3T 1E2, Canada; 2 Department of Medicine, McGill University, Montréal, QC H4A 3J1, Canada

**Keywords:** nucleocapsid protein, pan-virus, retrovirus, viral genomic RNA, liquid-liquid phase separation, biomolecular condensate, membraneless organelle, neurodegenerative disease, prion-like disordered protein domain, zinc finger motif, RNA-binding domain, zinc, copper

## Abstract

Liquid-liquid phase separation (LLPS) is a rapidly growing research focus due to numerous demonstrations that many cellular proteins phase-separate to form biomolecular condensates (BMCs) that nucleate membraneless organelles (MLOs). A growing repertoire of mechanisms supporting BMC formation, composition, dynamics, and functions are becoming elucidated. BMCs are now appreciated as required for several steps of gene regulation, while their deregulation promotes pathological aggregates, such as stress granules (SGs) and insoluble irreversible plaques that are hallmarks of neurodegenerative diseases. Treatment of BMC-related diseases will greatly benefit from identification of therapeutics preventing pathological aggregates while sparing BMCs required for cellular functions. Numerous viruses that block SG assembly also utilize or engineer BMCs for their replication. While BMC formation first depends on prion-like disordered protein domains (PrLDs), metal ion-controlled RNA-binding domains (RBDs) also orchestrate their formation. Virus replication and viral genomic RNA (vRNA) packaging dynamics involving nucleocapsid (NC) proteins and their orthologs rely on Zinc (Zn) availability, while virus morphology and infectivity are negatively influenced by excess Copper (Cu). While virus infections modify physiological metal homeostasis towards an increased copper to zinc ratio (Cu/Zn), how and why they do this remains elusive. Following our recent finding that pan-retroviruses employ Zn for NC-mediated LLPS for virus assembly, we present a pan-virus bioinformatics and literature meta-analysis study identifying metal-based mechanisms linking virus-induced BMCs to neurodegenerative disease processes. We discover that conserved degree and placement of PrLDs juxtaposing metal-regulated RBDs are associated with disease-causing prion-like proteins and are common features of viral proteins responsible for virus capsid assembly and structure. Virus infections both modulate gene expression of metalloproteins and interfere with metal homeostasis, representing an additional virus strategy impeding physiological and cellular antiviral responses. Our analyses reveal that metal-coordinated virus NC protein PrLDs initiate LLPS that nucleate pan-virus assembly and contribute to their persistence as cell-free infectious aerosol droplets. Virus aerosol droplets and insoluble neurological disease aggregates should be eliminated by physiological or environmental metals that outcompete PrLD-bound metals. While environmental metals can control virus spreading via aerosol droplets, therapeutic interference with metals or metalloproteins represent additional attractive avenues against pan-virus infection and virus-exacerbated neurological diseases.

## 1. Introduction

The recent re-classification of the eukaryotic cellular phenomena of phase separation of protein condensates as the underlying mechanism creating membraneless organelles (MLOs) for cellular compartmentalization is initiated by a liquid demixing program. Liquid-liquid phase separation (LLPS) is an evolved cellular survival strategy mediating stress-triggered environmental sensing and also nucleates the cellular self-assembly processes of biomolecular condensates (BMCs) required for many cellular processes, including signaling, cytoskeletal organization, and transcriptional regulation [1,2,3,4,5]. When exacerbated by a chronic stimulus including cellular stress, LLPS gives rise to the assembly and persistence of stress granules (SGs) associated with pathological disease onset [6]. LLPS also gives rise to aggregates found in cells derived from patients with neurodegenerative diseases [7]. A fundamental principle underlying biological molecules undergoing LLPS is multivalency and their capacity to simultaneously interact with multiple nucleic acids and proteins [8]. Indeed, self-aggregating mutated proteins that are hallmarks of neurological diseases, including SOD1, G3BP1, TIAR, TIA-1, DDX6, TDP-43, FUS/TLS, Tau, Amyloid β (Aβ), and hnRNP proteins [9], are also characterized as RNA-binding proteins, as helicases or chaperones, and as components of SGs or processing bodies (PBs) [10,11]. These proteins can be Zn- [12,13,14,15] or Cu- [15,16,17,18,19,20] regulated or regulating, and can undergo LLPS due to their low-complexity, intrinsically disordered prion-like disordered protein domains (PrLDs) [21,22]. These proteins also represent commonly used markers of SG assembly blockade imposed by numerous viruses that also co-opt them towards their replication [11,23,24].

Many different viruses employ LLPS to engineer BMCs used for their replication. Various virus family-centric terms and functions are used to describe these ‘viral replication compartments’ (VRCs; RCs), ‘virosomes’, ‘virus factories’ (VFs), ‘viroplasm’, ‘mini-organelles’, ‘inclusion bodies’, and ‘negri bodies’ (NBs) [25,26,27,28,29,30]. NB viral factories described for rabies virus (RABV) and vesicular stomatitis virus (VSV) have been characterized as originating from LLPS [31,32], and RABV, VSV, Ebola, and measles virus proteins undergo PrLD-dependent LLPS for host-defense shielding [31,32]. Viral proteins from divergent viruses, including influenza A, hendra, and herpes simplex, utilize LLPS to generate “liquid organelles” assisting replication [33,34,35]. Across divergent virus families, association of viral nucleoproteins (N) and viral RNA (vRNA) promotes LLPS [34,36,37,38], and association of zinc fingers (ZnFs) and RING finger proteins establishes virus factories, VLPs, and inclusion bodies [39,40,41,42,43,44,45]. As the ability of proteins to phase-separate stems from their PrLDs, large in silico meta-analyses demonstrate disproportionately higher degrees of PrLDs in viral proteins relative to eukaryotic proteins [46]. PrLDs are speculated to facilitate multiple inter-protein interactions maximizing the ability of viral protein condensates to compete for host proteins required for replication [47]. High degrees of disorder are also speculated to compromise vaccine design by providing viruses with immune-evading ‘shapeshifting’ abilities [48]. Degrees of disorder of viral proteins have recently been used to accurately predict environmental resistance, persistence as aerosol droplets, and transmission routes of coronaviruses, including SARS-CoV-2 [49].

We have recently demonstrated that the Nucleocapsid (NC) domain of HIV-1 pr55^Gag^ promotes Gag and Capsid (CA) domain-resistant SGs causing translational arrest [50,51], supporting a role for it providing an equilibrium between SG assembly and disassembly during HIV-1 replication [52,53]. More recently, we have shown that NC is produced in cells by active HIV-1 protease (PR) prior to virus budding [54]. The Zn^2+^-dependent HIV-1 NC domain is responsible for the positioning, trafficking, and packaging of the viral genomic RNA (vRNA) during virus assembly [55], and NC rapidly condenses into Zn^2+^- and ZnF-dependent LLPS condensates in vitro and in living cells [54]. Measles virus nucleocapsid (N) condensates by LLPS are also triggered by RNA and promote virus assembly [56]. In silico methods mapping conserved overlapping PrLD and ZnFs across pan-retrovirus Gag proteins and observations that full-length Gag and numerous retrovirus NC proteins undergo Zn^2+^-dependent LLPS support a pan-retrovirus-wide model of virus assembly dynamics primed by LLPS [54]. The SARS-CoV-2 N protein has also most recently been shown to phase-separate with viral RNA in a Zn^2+^-dependent manner [57,58,59,60,61].

PrLD-containing proteins are central to dynamic protein interaction network coordinating hubs [62]. Crystallization of protein–RNA complexes has historically been particularly difficult due to high conformational flexibility by PrLDs [63], these causing LLPS during protein crystallization, with no further characterization of resulting aggregates and gels generally considered to be disordered phases [64]. The lowering of free energy by liquid droplet formation during crystallization experiments should be considered in future BMC-targeting drug design [65,66]. Specific amino acids within PrLDs that mediate dynamic and “fuzzy” RNA-binding interactions enable flexible nucleic acid scanning for binding specificity that correctly distorts nucleic acids for downstream activities [67].

Indeed, a core requirement of replication and budding of bona fide infectious virus particles is the specific binding of structural viral proteins to their cognate vRNA. Since vRNA binding for retrovirus Gag proteins is dependent on Zn^2+^ and ZnF motifs within PrLDs [54], we have used in silico methods to map PrLDs, ZnFs, and RNA-recognition motifs (RRMs; RNA-binding domains, RBDs) across ‘nucleocapsid’, ‘nucleoproteins’, and other vRNA-binding proteins of viruses that were selected on the basis of their historical ranking of danger to human health. We observe conservation in positioning and juxtaposition of PrLDs, ZnF, and RRMs across divergent viruses, suggesting that a Zn^2+^-dependent NC condensate model may be extended to represent a fundamental underlying mechanism nucleating virus replication, and an untapped avenue for pan-virus pharmacological targeting. To elucidate a common mechanism controlling and linking LLPS, SGs, neurological aggregates, and virus assembly dynamics, we performed literature surveys demonstrating that these assemblies are promoted and inhibited by cellular, physiological, and environmental exposure to Zn^2+^ and Cu^2+^. Our findings are supported by reports that modulation in Cu/Zn ratios and altered expression of proteins maintaining cellular and physiological ion homeostasis are hallmarks of both virus infections and neurological diseases. We and others have contributed to the greater body of literature promoting the theory that towards their own replicative benefit, underlying virus infections induce neurocognitive disorders and may exacerbate neurological diseases [68,69,70,71,72]. We extend this concept, demonstrating that viruses alter metal ion homeostasis and Cu/Zn ratios, creating cell-clogging, insoluble pathological prion-like aggregates of proteins otherwise undergoing LLPS supporting normal cellular functions.

## 2. Materials and Methods

### 2.1. Informatics

Prion-Like Amino Acid Composition (PLAAC; http://plaac.wi.mit.edu/), Predictor of Natural Disordered Regions (PONDR; http://www.pondr.com/) [73], and the MobiDB database of protein disorder and mobility annotations (http://mobidb.bio.unipd.it/) were used to identify and validate the positions of PrLDs in viral proteins. FASTA input sequences were obtained from the NCBI protein sequence database and analyzed using software default parameters, with VLXT and VSL2 output styles selected for PONDR, and regions having a >0.8 PONDR Score were mapped using Adobe Illustrator software. Mapped ZnF and RRM placements were determined using the NCBI protein sequence database, from supporting references listed in Table S2, or were identified using MOTIF Search (https://www.genome.jp/tools/motif/) and Prosite (https://prosite.expasy.org/) [74], which were validated by examining sequences of known RNA-binding lysine, arginine, glycine, and histidine residues [75]. Phylograms organizing proteins according to virus families were generated using phyloT (http://phylot.biobyte.de) [30] and Interactive Tree of life (ITOL; http://itol.embl.de) [76].

### 2.2. Data and Code Availability

This study did not generate new datasets or code. Protein sequences were obtained from the NCBI Reference Sequence Database, with Genbank IDs listed in Table S2.

## 3. Results

### 3.1. Juxtaposed PrLDs, ZnFs and RRMs in the Most Deadly of Viruses

Disordered metal-binding regions of proteins can be stabilized upon ion-binding for gain of structure and function [77]. We hypothesized that pan-virus assembly and budding and maintenance of infectious particles within aerosol droplets is nucleated by phase separation events initiated by viral protein PrLDs gaining structure to bind vRNAs via metal loading of their juxtaposing RRM ZnFs. We surveyed the literature to generate a list of the most dangerous human viruses historically responsible for the greatest number of mortalities in current and past epidemics and pandemics (Table S1).

We were interested to find which other viral proteins with functional equivalence to HIV-1 Gag in vRNA-binding and encapsidation and viral capsid architecture also possessed juxtaposing PrLDs, RRMs, and ZnFs, inducing metal-dependent condensates. In some cases where identification of functional orthologs of Gag was complicated by diverging viruses having vastly different replication intermediate steps, we broadened our inclusion criteria to accept viral proteins that bind viral RNAs, or viral proteins possessing characterized ZnFs.

**Figure 1:**
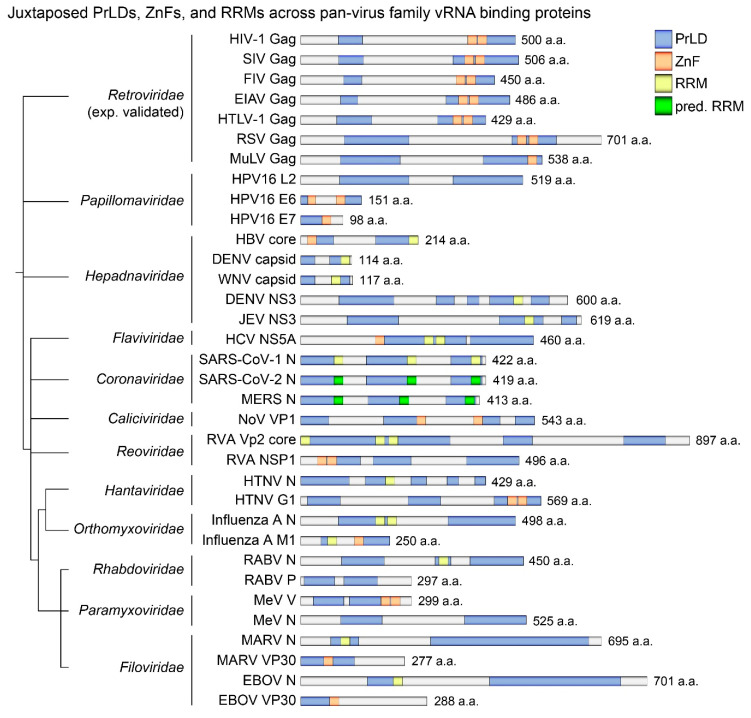
Phylograms of pan-virus proteins containing juxtaposed PrLDs, ZnFs, and RRMs. Members of distantly linked virus families were imported into phyloT (http://phylot.biobyte.de) [30] and ITOL (Interactive Tree of life; http://itol.embl.de) [76] to render phylogeny trees. Predictor of Natural Disordered Regions algorithm software (PONDR; http://www.pondr.com/) [73] with VLXT and VSL2 and default settings was used to map locations of PrLDs on viral proteins, analyzed using FASTA sequences, which were obtained from the NCBI protein sequence database. Predicted PrLDs (blue) having a >0.8 score were scaled for presentation using Adobe Illustrator. ZnF (orange) and RRM (yellow) placements were determined from the NCBI protein sequence database and the literature, and in other cases, were identified using MOTIF Search (https://www.genome.jp/tools/motif/) and Prosite (https://prosite.expasy.org/) [74], which were validated by examining sequences of known RNA-binding lysine, arginine, glycine, and histidine residues [75]. Experimentally validated (i.e., exp. validated) Retroviridae proteins included are those that undergo NC-, ZnF-, and Zn^2+^-mediated liquid-liquid phase separation (LLPS) [54]. PrLDs, prion-like domains; ZnFs, zinc fingers; RRMs, RNA-recognition motifs; pred. RRMs, predicted RNA-recognition motifs; a.a., amino acid; HIV-1, human immunodeficiency virus-type 1; SIV, simian immunodeficiency virus; FIV, feline immunodeficiency virus; EIAV, equine infectious anemia virus; HTLV-1, human T-cell leukemia virus 1; RSV, Rous sarcoma virus; MuLV, murine leukemia virus; HPV16, human papillomavirus 16; HBV, hepatitis B virus; DENV, dengue virus; WNV, West Nile virus; JEV, Japanese encephalitis virus; HCV, hepatitus C virus; SARS-CoV-1, severe acute respiratory syndrome coronavirus 1; SARS-CoV-2, severe acute respiratory syndrome coronavirus 2; MERS-CoV, Middle East respiratory syndrome-related coronavirus; NoV, norovirus; RVA, rotavirus A; HTNV, Hantaan orthohantavirus; influenza A, influenza virus; RABV, rabies lyssavirus; MeV, measles virus; MARV, Marburg virus; EBOV, Ebola virus.

To map proximities of viral protein PrLDs, RRMs, and ZnFs, NCBI protein database amino acid sequences were analyzed using PONDR and PLAAC algorithms [73,78,79,80] (Table S2). Wherever possible, predicted viral PrLDs were validated by previous studies, while others were validated by the MobiDB database [81]. The accuracy of predictive software programs was formerly tested as described [54]. These bioinformatic analyses revealed that many of these viral structural proteins contain two conserved PrLDs of similar length and location relative to protein length formerly observed for pan-retrovirus Gag proteins (Figure 1) [54]. In cases where RRM or ZnF positions were not provided by the database, extensive literature searches were performed to map their characterized locations (see refs in Table S2). Due to differences in the way many viruses replicate, and in some cases due to virus family-centric literature, many of these proteins could only be classified as functionally equivalent HIV-1 Gag and NC domain orthologs from their disorder and Zn^2+^-dependence, or from their propensity to multimerize, phase-separate, and bind vRNAs [55,82,83,84,85,86,87,88,89,90]. Other orthologous selected proteins including non-structural proteins are previously characterized as being disordered early replication intermediates that bind vRNAs for assembly and encapsidation, or as providing structure, stability, resistance, and infectivity to virus cores [49,75,91,92,93,94,95,96,97,98,99,100,101,102,103,104]. Finally, other informative reports have described some selected orthologous viral proteins as disordered vRNA or nucleoprotein chaperones [55,105,106,107,108].

The combinatorial features of PrLDs, RRMs, and ZnFs towards a chaperoned RNA-LLPS model nucleating virus assembly that we have recently described for HIV-1 is one that is dependent on the Gag NC domain [54] and may also be mirrored by other distantly related viruses. For example, disordered N protein and phosphoprotein (P) are features of well characterized mononegaviruses utilizing phase separation towards replication [32,109,110,111,112,113,114,115,116], where P acts as a chaperone to delay N RNA-binding promiscuity to advantage vRNA binding specificity and downstream LLPS leading to virus biogenesis [56]. Indeed, PrLDs and high surface charges typical of RNA-binding motifs are associated with chaperone interaction protein hubs facilitating interaction with multiple proteins within interaction networks [117,118]. Intriguing research suggests that highly disordered RNA chaperones were among the earliest proteins to evolve, with their PrLDs providing solubilization and entropic exclusion effects and the bypassing of energy consuming iterative annealing activities [119,120]. Plant virus movement proteins (MPs) are also disordered and possess a wide range of functions, such as interacting with viral proteins and vRNA to form ribonucleoprotein complexes facilitating cell-to-cell and long-distance movement of the viral genome within the plant body [121,122]. The cysteine-histidine-rich region of cucumber mosaic virus MP contributes to plasmodesmal targeting and Zn^2+^ binding and pathogenesis. This virus’ ZnFs juxtapose C-terminal PrLDs as we have observed for other viral vRNA-binding proteins, while ZnF mutants have attenuated virus infectivity [123].

High degrees of disorder thus represent an evolutionary asset providing ancient viruses, encoded by very little genetic material, a replicative advantage and protection against host-factor interference or immune recognition. Indeed, viral nucleocapsid (nucleoproteins) have also frequently been characterized as viral RNA or protein chaperones. Since RBD-containing PrLD proteins are those that nucleate SGs [118], to replicate undetected, viruses may have evolved SG-blocking mechanisms that they would otherwise themselves be inducing. Results of our PrLD and RNA-binding domain mapping across viral NC, N, or CA proteins (all referred to here as NC proteins) from broadly different genera provide conclusive evidence that these have overlapping or juxtaposing PrLDs, RRMs, and ZnFs that may engineer BMCs towards virus biogenesis.

### 3.2. Metal Ion Binding Competition for ZnFs and RRMs Alters Protein Aggregate Stoichiometry

In our previous work, we observed that although Spumaviruses do not have ZnFs, these, nevertheless, have an abundance of RRMs mapping to the same approximate positions juxtaposing conserved C-terminal PrLDs found for all retroviruses [54,124]. Therefore, it is expected that Spumavirus RRMs provide the same function as ZnFs to retroviral Gag NC domains. In this work, we also find that NC vRNA-binding proteins from different viruses commonly have either ZnFs or RRMs juxtaposing PrLDs. Just as Spumavirus RRMs replace ZnFs for vRNA binding, Zn^2+^ binds to RRMs of other cellular proteins that phase-separate and assemble into SGs [13]. RRM regions have also been identified as the molecular determinants of phase-separating eukaryotic Pab1 protein [1].

Viral proteins that undergo phase separation bind vRNAs via RRMs or ZnFs within their PrLDs. Many cellular RNA-binding proteins (e.g., helicases LAF-1 and DDX4 and hnRNPA1) have arginine-rich, positively charged sequences (e.g., RRMs, RGG boxes, SR repeats) that drive LLPS in an ionic strength-dependent manner [7,125]. Positively-charged lysine, arginine, and histidine residues interact with ribose and phosphate moieties of nucleic acid, mediating the vRNA encapsidation process by viral NC proteins [75]. ZnFs, on the other hand, provide negatively charged cysteine residues that, upon Zn^2+^ binding, mediate conformational changes of NC proteins, permitting binding to coiled nucleic acid grooves.

Among the many roles of metal ions in biological processes, these bridge interactions between distant residues of protein domains mediate protein–ligand interactions and serve as nucleophilic catalysts of enzymatic active sites. Although many biological processes are historically accepted as metal-ion dependent, a growing body of knowledge also demonstrates that metals can be interchangeable with certain cellular mechanisms. Metals are an integral part of viral proteins and play important roles in their survival and pathogenesis. Zn^2+^, Cu^2+^, and magnesium (Mg^2+^) are the most common metal ions binding to viral proteins and participate in strand transfer during reverse transcription of the vRNA, nucleic acid annealing and integration, transcription, and vRNA maturation [126]. The importance of metal ions in the survival and pathogenesis of many viruses cannot be understated, and structural studies for metal binding to viral proteins are useful for design and development of viral inhibitors [126].

Tight control of divalent ion concentrations and homeostasis is most critical to cellular health and longevity [127]. Rapidly changing cellular conditions, including stress, cause ion fluxes, coinciding with formation and dissolution of MLOs and peptide-RNA condensates [128]. Whereas divalent ions, such as Zn^2+^, positively influence phase separation [12], others (e.g., Mg^2+^, Ca^2+^) negatively influence PrLD–RNA interactions and reduce LLPS, as does the metal chelator EDTA [128]. Although Zn^2+^ is the presumed metal binding ZnFs, ZnFs also coordinate numerous other metal ions (reviewed in [129]). As the third most abundant metal following iron (Fe) and Zn in eukaryotic cells, copper (Cu) is of particular interest because its binding to classical and non-classical ZnFs, including HIV-1 NCp7, is thermodynamically favored over the binding of other metals, including Zn^2+^ [130]. Copper-binding, however, does not induce secondary structure of ZnFs, rendering them non-functional and compromising their ability to bind to DNA or RNA [129]. Cu^2+^ has also been demonstrated to bind to glycine-rich stretches of RRM-containing proteins, abrogating their abilities to bind to RNAs and impairing their general cellular functions [131].

Aβ protein has been extensively studied as a prime causative agent of neurodegenerative disease deposits (i.e., protein aggregates, plaques). Importantly, a number of different metal ions associate with monomeric Aβ to induce its aggregation but individually cause measurable variations in the types of Aβ deposits they induce [132,133]. Indeed, it is well established that Cu^2+^-binding prevents Aβ protein β sheet conformation and aggregation [132,134] and similarly inhibits aggregation of other amyloidogenic peptides [135,136]. Most intriguingly, compared to other metals, Cu^2+^ binding to Aβ is more kinetically favored, and Cu^2+^ can bind to previously self-aggregated Aβ [18]. Zn^2+^-binding, on the other hand, induces spherical Aβ structures different from β sheet structures [137]. Early evidence demonstrated that these two metals play opposing functions, with even low Cu^2+^ concentrations inhibiting Zn^2+^-induced aggregates [138]. It was later shown that Cu^2+^ and Zn^2+^ bind to the same Aβ histidine residues, where Zn^2+^ precipitates at least two peptides to induce spherical structures, whereas Cu^2+^ outcompetes Zn^2+^ to inhibit intra-protein contacts [139]. Indeed, from numerous metal ions tested (including Cu^2+^), Zn^2+^ alone could induce Tau protein condensates, another prime causative agent of neurodegenerative disease deposits [14]. The cellular prion protein also binds to both Zn^2+^ and Cu^2+^, with both metals inducing structural changes and decreased solubility [140] and differentially affecting its fold variants [141,142], where Cu^2+^-binding induces protease resistant variants [143,144]. Relevant to viruses existing in cells and tissues, in plasma and in external environments, studies comparing competitive binding of Cu ions to peptides containing both cysteines (e.g., ZnFs) and arginines (e.g., RRMs), Cu ions were found to preferentially bind to arginine, lysine, and histidine in the gas phase [145]. Indeed, Figure 1 demonstrates that viral proteins possess positively charged PrLD-juxtaposed RRMs and ZnFs. With metal ions controlling and maintaining the structure and functions of viruses [126], modulations in cellular Cu/Zn ratios have the potential to influence viral protein conformations and interactions. A better understanding of how these two metals affect virus biogenesis, and design and testing of endogenous or environmental metal-targeting agents, may promote establishment of pan-virus therapeutics or biocides.

### 3.3. How Zn^2+^ and Zn^2+^-Chelation Both Cause Loss of Viral Capsid Integrity

An interesting conundrum is exposed in the examination of the requirement of viruses for Zn^2+^ versus circulating Zn^2+^ levels in virus-infected patients. Indeed, Zn^2+^ is critical for innate and adaptive immunity [146] and its deficiency is the most prevalent micronutrient abnormality in HIV-1 infected individuals [147,148,149,150], even persisting in patients treated with anti-retroviral therapy (ART) [151,152,153]. Zn^2+^ deficiency correlates with diminished CD4^+^ T cells, high viral loads, AIDS, and mortality [149,151,153,154,155,156,157,158,159], and its supplementation delays disease progression [147,160,161,162,163,164]. However, while infected patients experience Zn^2+^ deficiency, virus replication requires Zn^2+^ [126,165].

Zn^2+^ is the most common viral protein cofactor assisting numerous replication processes of both RNA and DNA viruses [165]. In addition to its requirement for the myriad of functions performed by HIV-1 NC [166], Zn^2+^ also confers proper folding and varied functions to HIV-1 Integrase and Tat and Vif proteins [165,167,168,169,170,171,172,173,174,175,176]. From their high conservation among all viral clades and their many essential functions during replication, NC ZnFs are a primary target for the continued development of potent and specific clinical Zn^2+^ ejectors [177,178,179,180,181,182,183,184,185,186,187,188,189,190,191,192,193,194].

Zn^2+^ deficiency is also common to infection by numerous other viruses, and Zn^2+^ supplementation as an antiviral therapy has been clinically tested against many divergent viruses, including herpes simplex virus (HSV), rhinovirus (RV), influenza, human papillomavirus (HPV), HIV-1, and hepatitus C virus (HCV) [160]. Zn^2+^-deficient plants are also more susceptible to diseases. Zn^2+^ deficiencies result from infection by turnip yellow and tobacco mosaic viruses, and plants supplemented with Zn^2+^ have ameliorated responses to pathogenic diseases [195,196,197]. Zn^2+^ was first reported to inhibit RV in 1974 [198], and zinc salts and lozenges have since been applied against the common cold and influenza [199]. Numerous in vitro studies have since demonstrated the potent antiviral efficacy of Zn^2+^ against coronavirus, encephalomyocarditis virus, foot and mouth disease virus, HCV, HSV, HIV-1, HPV, Rous sarcoma virus (RSV), Semliki Forest virus (SFV), Sindbis virus, vaccinia virus, varicella-zoster virus, HEV, and arteriviruses [160,200,201,202]. Unfortunately, the required antiviral Zn^2+^ concentrations tested exceed safe physiological ranges associated with clinical testing [203,204], promoting developments of less toxic compounds, including zinc oxide nanoparticles (ZnO-NPs), demonstrating antimicrobial activities against influenza virus H1N1 and HSV-1 [205,206].

It is puzzling that Zn^2+^ both promotes and inhibits virus replication, while its deficiency is associated with increased viral load, suggesting that there are major gaps in our understanding of how this metal influences viruses. While Zn^2+^ is required for virus replication, its chelation inhibits viral proteins (HPV E6 protein, flavivirus NS5A protein) and virus replication (HPV, dengue virus (DENV), and Japanese encephalitis virus (JEV)) [207,208,209,210]. Despite severe toxicities associated with experimental Zn^2+^ chelators, such as TPEN (N,N,N′,N′-tetrakis(2-pyridinylmethyl)-1,2-ethanediamine) and 1,6-hexanediol, other drug classes with better safety profiles, including bananins, may be effective against HIV-1 and other viruses, including coronaviruses [211,212]. Zn^2+^ ionophores, such as pyrithione and chloroquine derivatives, among others, have also recently been shown to inhibit diverse groups of viruses, such as HSV, DENV, and SARS-CoV-2 [210,213,214].

Another piece of the puzzle may involve Zn^2+^- and Cu^2+^-regulating and -regulated cysteine-rich metallothioneins (MTs) [215]. Despite the precise mechanisms for these remaining elusive, upregulation of MTs is observed in response to virus infections by measles virus (MeV), influenza, HIV-1, HCV, and coxsackie virus [160]. Metalloproteins and Zn^2+^ imbalances are also present in human cancers and are also linked to regulation of HPV infection [216]. As with HIV-1, pathological HPV16 strains integrate into host DNA, but of cervical keratinocytes, persisting and immortalizing these into aggressive cervical malignancies via expression of HPV E6 and E7 proteins [217,218]. Notably, the coordination of Zn^2+^ by the HPV16 E7 protein produces a compact environment leading to its self-assembly into spherical oligomers similar to those found in amyloids, whereas Zn^2+^ depletion results in loss of its aggregation [219,220,221]. HPV E6 protein also forms Zn^2+^-dependent soluble agglomerates, whereas the metal chelating agent EDTA stabilizes its monomeric form and destabilizes existing agglomerates [89], and where Cu^2+^ complexes also cause E6 aggregation, inhibiting its function [222]. Recent work has demonstrated that the keratinocyte-derived body’s epidermal barrier is formed by the filaggrin protein [223], a protein that phase-separates and that has been found to bind Cu^2+^ and be regulated by Zn^2+^ [224].

Confounding observations that both Zn^2+^ and Zn^2+^-chelation both negatively affect viruses may be explained by differing research aims or experimental models from past reports. If virus biogenesis and assembly is Zn^2+^-dependent, then pre-formed virus capsid structure may be challenged by higher external concentrations of Zn^2+^ outcompeting virus-internal Zn^2+^, just as Zn^2+^ chelation would. In addition, if metal ions ultimately regulate virus core assembly (and stability) nucleated by metal-induced condensates during late stages of virus replication, then virus cores at early stages of reinfection should also be susceptible to metal ions or their chelation. Indeed, another effective antiviral agent, the zinc-finger-reactive disulfide NSC20625, ejects Zn^2+^ from JUNV Matrix protein RING finger motif, causing incomplete virion uncoating and release of NC into the cytoplasm [225]. Zn^2+^ bound to highly conserved ZnFs of influenza virus M1 matrix proteins is described as the determining factor of conformational transition of capsids in acidic environments, leading to their uncoating [226]. Similar to HIV-1 Gag, M1 is highly disordered, contains both RRMs and ZnFs (Figure 1), and is the most abundant viral protein, responsible for both recruiting newly synthesized RNP cores from the nucleus for encapsidation and for maintaining the virion structure [224]. Upon infection of cells, the influenza M2 protein opens ion channels to permit flux into virions, and resulting pH flux and Zn^2+^ cause virus uncoating by destabilizing Zn^2+^-bound M1 protein [226]. Poliovirus capsid is also similarly destabilized by Zn^2+^, leading to increased virus permeability [227].

To determine how viruses are affected by exogenous Zn^2+^, ultimately regulating virus core assembly and stability, susceptibility of cell free viruses to environmental metal ions or their chelation should be evaluated. Indeed, virucidal agents ejecting Zn^2+^ from NC to inactivate HIV-1 have been developed for use as topical microbicides [185]. Non-toxic, hybrid cured surface coatings containing Cu^2+^ and Zn^2+^ also show virucidal activity against HIV-1 and other enveloped viruses [228]. Thus, both environmental Zn^2+^ ions and Zn^2+^ chelators inactivate viruses. Landmark studies relevant to the current COVID-19 pandemic tested a range of surfaces to find that copper alloys and Cu/Zn brass surfaces inactivated replication and propagation abilities of SARS-CoV [229,230] and inactivated norovirus by disrupting capsid integrity [199]. Norovirus VP1 protein is also populated by PrLD-associated ZnFs (Figure 1). Indeed, Zn^2+^ is found to synergize with Cu^2+^ in surfaces and to increase efficacy of brass surfaces with lower percentages of Cu^2+^, suggesting that these ions destabilize capsids for viral genome destruction [199,231]. Importantly, it is reported that although partial disorder around the dynamic loop regions limits precise positioning, the VP1 of the disordered P2 spike region of the norovirus outbreak strains binds to a Zn^2+^ ion that affects shell stability [232]. Indeed, SARS coronavirus’ NC (N) proteins have a high degree of disorder, self-associate, and exhibit promiscuous binding to vRNA [95,233]. Importantly, phase separation of the SARS-CoV-2 N protein and RNA is promoted by Zn^2+^ and is also influenced by Cu^2+^ [61]. Intrinsically disordered regions of the SARS-CoV-2 spike (S) protein have been identified to offer a selective advantage for its binding affinity to the Zn^2+^ metallopeptidase angiotensin-converting enzyme 2 (ACE2) entry receptor, and high numbers of Zn^2+^-binding cysteine residues within these regions may also contribute to increasing binding affinity of the S protein to ACE2 [234,235,236].

Despite the many instances demonstrating that Zn^2+^ supports capsid assembly and structure across viruses through its interaction with ZnFs within PrLDs, difficulties in identifying effective therapeutics interfering with ZnFs or PrLDs for the elimination of virus condensates lead us to consider additional ways to control Zn^2+^-dependent viral condensates by examining upstream physiological or cellular regulating processes. From reports above describing Zn^2+^ binding site competition by other ions, such as Cu^2+^, that interfere with the establishment of condensates, below we describe alternative routes to control pathologic forms of LLPS, as supported by ancient evolutionary protein chaperone pathways.

### 3.4. Environmental Cu^2+^ as a Means to Control Viruses

Copper (Cu^2+^) has been used to disinfect fluids, solids, and tissues for centuries. Cu^2+^ was discovered by the ancient Greeks in the time of Hippocrates (400 BC), who used it to purify water and treat pulmonary disease. For their anti-fouling properties, Cu^2+^ vats were used to store holy water from the Ganges River, and Cu^2+^ strips were also used to construct ship hulls by the early Phoenicians. By the 18th century, Cu^2+^ was widely accepted for clinical treatment of mental disorders and pulmonary diseases. Both early American pioneers and Japanese soldiers of WWII placed Cu^2+^ coins to sterilize their drinking water, and NASA Apollo flights used Cu^2+^-based water sterilizing systems. Cu^2+^ has also historically been used by Africans and Asians to treat skin diseases [237]. Indeed, physiological Cu^2+^ is highly abundant and safe, with 1 mg consumed daily and excess copper released by excretion. More modern demonstrations of Cu^2+^ safety come from widespread and long-term use of Cu^2+^-based intrauterine devices (IUDs) to which human tissues have low sensitivity [238,239]. Meta-analyses have provided evidence that use of Cu^2+^-based IUDs correlated with 50% lower incidence of cervical cancers [240].

Copper is a well-established biocide used against viruses and many other pathogens. In 1964, Cu^2+^ was first shown to inactivate bacteriophages [241], followed by reports of Cu^2+^ inactivating infectious bronchitis virus in 1971 [242]. In 1974, the effect of Cu^2+^ on poliovirus RNA was proven to be proportional to its concentration, as well as that most amino acids except cysteine had a protective effect against Cu^2+^ [243]. In 1992, Cu^2+^ was found to inactivate enveloped or non-enveloped and single- or double-stranded DNA or RNA viruses (i.e., phi X174, T7, phi 6, Junin, and HSV) [244,245]. During this time, cellular and cell-free HIV-1 was also shown to be inactivated by Cu^2+^ concentrations lower than that required for inactivation by ethanol and where Cu^2+^ preserved cell viability while completely inhibiting formation of syncytia and virus production [246]. More precisely, incubation of reconstituted HIV-1 NC protein with Cu^2+^ caused its cysteine-dependent oxidation [247]. HIV-1 protease is also inactivated by Cu^2+^ in a cysteine-dependent manner, which was most notably found to cause its aggregation [237].

The use of copper in free flow filters deactivates HIV-1 and West Nile virus, reducing infectious titers of these viruses by 5 to 6 logs [248]. Cu^2+^-containing filters also effectively neutralize HIV-1 in medium and breast milk and reduce cell-associated HIV-1 in a dose-dependent manner [249]. Cu^2+^-containing filters also reduce infectious viral titers of several DNA and RNA viruses, including YFV, influenza A virus, MeV, RSV, adenovirus type 1, and cytomegalovirus [250]. Likewise, Cu^2+^ in water pipes synergize with low levels of free chlorine has proven effective in inactivation of poliovirus, bacteriophage MS-2, hepatitis A virus, human rotavirus, and human adenovirus [237,251]. The International Copper Association has found that Cu^2+^ reduced survival and infectivity of waterborne viruses poliovirus, coxsackie virus types B2 and B4, echovirus 4, and simian rotavirus SA11 by 95% [237]. Finally, plant viruses, including the cucumber mosaic virus, are also subject to copper-dependent inactivation [252].

Non-toxic, hybrid cured coatings containing copper also have virucidal activity against HIV-1 and other enveloped viruses [228]. The risk of contaminated surfaces and the use of antimicrobial surfaces and materials sciences in high-risk environments have recently been highlighted by the COVID-19 pandemic [253]. Since preventative strategies are perhaps as important as discovering healing drugs or therapies, antimicrobial surfaces are truly poised to prevent the spread of many infectious agents retaining infectivity on surfaces. Cu^2+^ alloys can rapidly and effectively kill a wide range of microbial pathogens at a range of temperatures and under various conditions of humidity [199]. Clinical trials incorporating copper surfaces in hospital wards found reductions in the bioburden, and lowered infection rates from equipping rooms with just a few copper surfaces [254,255,256,257]. Incorporation of Cu^2+^ alloys is useful in preventing secondary transfer from surfaces in clinical facilities and other closed environments, including long term care facilities, public washrooms, cruise ships, and casinos [199]. As with other viruses, SARS-CoV-2 is destroyed by Cu^2+^ in hours, while it can remain infectious on other surfaces for days [230]. This has spurred the development of copper stickers and surface coatings demonstrated to inactivate 99.9% of viral titers of SARS-CoV-2, EBOV, and Marburg virus (MARV) [258]. Manufacture of copper face masks to eliminate aerosol transmission events is also in development, as earlier suggested for control of influenza [259], also inactivated by Cu^2+^ surfaces [260]. Textile fibers, latex, and other polymers impregnated with Cu^2+^ also possess broad-spectrum anti-microbial and antiviral properties [248].

### 3.5. Endogenous Cu^2+^ as a Means to Control Viruses

A number proteins are bound by Cu^2+^ for their functions, while others bind to and control the uptake and delivery of Cu^2+^ for cellular and physiological processes. Cytosolic Cu-Zn-superoxide dismutase (SOD-1) is a ubiquitous cytosolic homodimeric isoenzyme that scavenges and catalyzes the dismutation of superoxide radicals [261]. Several variants of familial neurodegenerative disorder amyotrophic lateral sclerosis have been linked with SOD-1 mutations, affecting metal-binding sites occupied by Cu^2+^ and Zn^2+^ and leading to distorted SOD-1–SOD-1 interactions, leading to formation of insoluble aggregates described above [262]—a phenomenon that can also be propagated from cell to cell [263]. Intriguingly, many large DNA viruses, including chordopoxviruses [264], entomopoxviruses [265], and baculoviruses [266], encode catalytically inert SOD-1 decoy homologs. Most poxvirus-encoded SOD-1 homologs belong to one of two well conserved structural classes comprising either of the orthopoxvirus genes having undergone extensive evolutionary deletion mutagenesis events [267]. All characterized poxvirus SOD-1 homologs to date are catalytically inactive [268] but may retain their metal-binding capacities, perhaps functioning as metal donors or chelators, with some mutants binding to the SOD-1 Cu^2+^ chaperone [264]. Modulated SOD-1 expression and activity has also been observed from infection by respiratory syncytial virus and influenza A virus [269,270]. Cu/Zn-SOD activity is also modulated in plants infected by viruses [271].

Ceruloplasmin (CP) is another Cu^2+^-binding glycoprotein [272], mostly produced and secreted by hepatocytes, and represents the largest physiological contributor of Cu^2+^, accounting for more than half of plasma Cu^2+^ [273,274,275]. CP transports Cu^2+^ from the liver and delivers it directly to cells for the synthesis of 40–70% of Cu^2+^-binding proteins and enzymes of various organs and tissues [276,277]. A common hallmark of infection, irrespective of causative microbial agent (e.g., viral, bacterial, fungal), is a marked and progressive increase in serum Cu^2+^ attributed to CP [278,279,280], suggested to deliver Cu^2+^ to the sites of infection to attack invading pathogens with Cu^2+^ toxicity [281]. CP expression is downregulated by HBV replication and otherwise inhibits the production of extracellular virions by targeting its middle surface protein, without direct involvement in HBV replication, suggesting that CP may rather represent an important host factor targeting assembly and/or release of virions [282]. CP is also increased during the acute period of chronic recurrent HSV infection, is decreased during HSV remission [283], and remains increased in the plasma during remission periods of patients with severe forms of HSV [283]. CP is also associated with neurocognitive impairment in HIV-1 infected patients [284,285]. Increased CP expression is also observed in patients with amyotrophic lateral sclerosis (ALS) [286] and Alzheimer’s disease [287], where its cerebrospinal fluid levels predict cognitive decline and brain atrophy in patients with underlying Aβ pathologies [287] and where CP has been postulated to act to defend against neurodegenerative diseases [288]. These accounts suggest that modulation in CP expression, and thus modified physiological and cellular Cu^2+^ homeostasis, may exacerbate the LLPS underpinnings of viral and neurological diseases.

Imbalances in homeostasis of physiological metals are important disease biomarkers. Plasma Cu/Zn ratios represent a common clinical assessment for Zn^2+^ deficiencies associated with several diseases [289]. Elevated serum Cu/Zn ratio is indicative of nutritional deficiencies, oxidative stress, inflammation, and immune abnormalities [290], contributing to an increased risk of all-cause, cancer, and cardiovascular-related mortalities [291]. It also serves as a common biomarker of frailty linked with multiple-cause mortality in the elderly and of advancement of chronic disease [292,293] but also as a biomarker for children predisposed for numerous pediatric diseases, including vascular complications, cancers, and virus infections, and for neonates with early-onset congenital infections [294,295,296,297,298]. What leads to altered plasma Cu/Zn ratios is still, however, unknown [299]. Significantly raised serum Cu/Zn ratio is proportional to infection in HIV-1-infected patients relative to healthy subjects [300,301] and is a useful predictor of disease progression and mortality in HIV-1-infected patients [159]. Increased serum Cu/Zn ratios, in combination with diminished SOD-1 levels, are biomarkers that stratify progressing HTLV-1-infected patients [302].

Fatal neurodegenerative prion diseases are caused by proteinaceous infectious particles, or “prions”. Prion disease is caused by the accumulation of abnormally folded isoforms of the cellular prion protein (PrP, encoded by the PRNP gene), where the unstructured amyloidogenic region of PrP preferentially binds to Cu^2+^, inducing its beta-sheet conformation and aggregation [303]. Recent evidence demonstrates that PrP is antimicrobial, anti-viral, and also interacts with the antimicrobial Aβ protein [304]. Antimicrobial peptides (AMPs) are a large and diverse group of ancient proteins conserved between humans and primitive fish, pre-dating the adaptive immune system, with many of these binding to Cu^2+^ and nucleic acids [305,306]. PRNP and PrP are upregulated during virus infection by HIV-1, VSV, MLV, HCV, adenovirus 5, and Epstein–Barr virus [307,308,309,310,311], and in brains of HIV-1- and SIV-infected patients and macaques [312]. PrP is also upregulated in patients with Alzheimer’s disease, a neurological disease suspected to be exacerbated by viral infection [313,314]. Notably, the formation of the ‘scrapie-specific’ neurotoxic form of PrP (i.e., PrP^sc^) is induced by HIV-1 infection [304,315]. As demonstrated for other proteins, Cu^2+^- or Zn^2+^ -binding also induces PrP fold variants [141,142], suggesting that metals differentially influence PrP conformations and thus increase propensity for generation of pathological aggregates. Across the many different ions tested, the binding of Zn^2+^ and Cu^2+^ to PrP is accompanied by structural changes and decreased solubility, where Cu^2+^-binding causes protease resistant PrP conformations [140,143,144].

## 4. Discussion and Model Supporting Zn^2+^- and Cu^2+^-Mediated Control of Pan-Viruses

**Figure 2:**
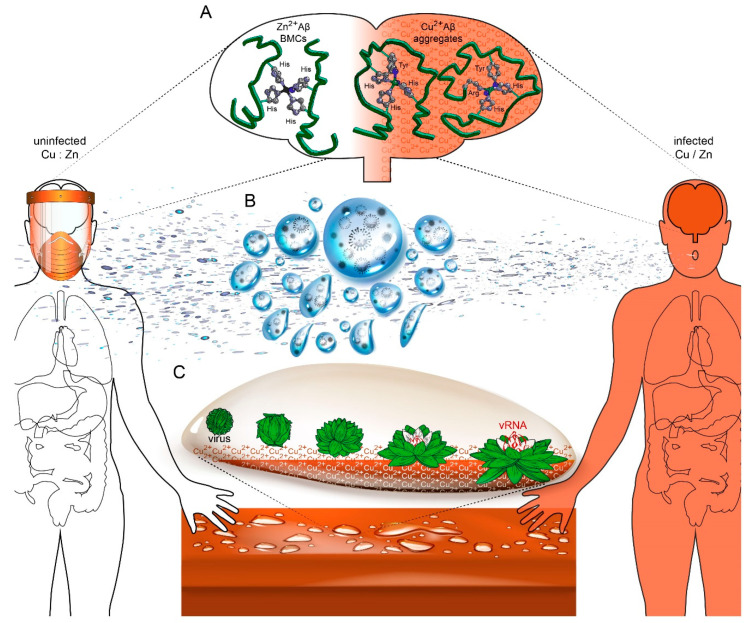
Model supporting virus exacerbation of neurological diseases via altered Cu/Zn ratios, and virus inactivation by Cu^2+^ coated surfaces. (**A**) An example of pathologic proteins causing neurological disease aggregates: differential folding conformations of Aβ protein, as influenced by competing Zn^2+^ and Cu^2+^, where Zn^2+^ outcompeting Cu^2+^ ion binding to prion and prion-like proteins causes their aggregation into insoluble plaques that are the hallmarks of neurological diseases. Zn^2+^ and Cu^2+^ homeostasis is altered by chronic virus infections and other diseases, generating an increased physiological Cu/Zn ratio. Although poorly understood, modified expression of numerous circulating and cellular metalloproteins and metal-ion carrier proteins by bodily defenses against underlying virus infections may lead to altered Cu/Zn ratios in an attempt to destroy viruses and the simultaneous promotion of Cu^2+^-associated protein aggregates. (**B**) Breath condensate plume from a coughing infected individual (right) to illustrate virus aerosol droplets, in which whole viruses may hold together as phase-separated condensates and which spread and are deposited onto surfaces such as counter tops, illustrated below. (**C**) Cu^2+^ causes the inactivation of viruses and the loss of virus capsid integrity. Cu^2+^ ions from Cu^2+^ coated surfaces may outcompete Zn^2+^ ions that are responsible for proper folding of PrLDs forming LLPS condensates, thereby causing premature virus uncoating and destruction if exposed vRNA. Altogether, the model supports the use of Cu^2+^-coated surfaces as potent pan-virus antimicrobial (image credits: modified images generated by Upklyak and Articular at www.freepik.com and [139]). Aβ, Amyloid β; Zn, zinc, Cu, copper; Zn^2+^, zinc ion; Cu^2+^, copper ion; Cu: Zn; copper-zinc homeostasis; Cu/Zn; increased copper to zinc ratio; vRNA, viral genomic RNA.

We were interested in gaining a better understanding of mechanistic intersections and fundamental features of proteins commonly associated with pan-viral engineered BMCs and neuropathologies. To propose a simplified model illustrating how viral infections may exacerbate neurological diseases, a bioinformatics-coupled meta-analysis system biology framework investigated how cellular, physiological, and environmental metals may govern both essential BMCs and pathological protein aggregates. The model illustrates how physiological Zn^2+^ and Cu^2+^ metal ions differentially influence formation of BMCs and protein aggregates of neurological diseases, and then extends to how viral infections induced metal imbalances may exacerbate neurological diseases (Figure 2A). Such investigations may lead to development of therapeutic avenues against a panoply of previously seemingly unrelated diseases. Additionally, pre-existing clinical tests profiling metal homeostasis could accompany other companion diagnostics for evidence-based treatments against viral and neurological diseases. Finally, in light of the economic impacts of a pandemic, coating metal agents could potentially reduce the spread of viruses while targeting drugs or vaccines are awaited.

The model first illustrates how virus infections cause increases in both Cu/Zn ratios and expression of ancient antimicrobial prion proteins, such as Aβ protein. For one, this suggests that an evolutionary conserved anti-viral mechanism promotes heightened Cu^2+^ bioavailability to inactivate viruses, possibly by outcompeting Zn^2+^-dependent processes during both early and late stages of virus replication. In early stage virus binding, entry, and uncoating, high Cu^2+^ concentrations may prematurely disrupt pre-formed virus capsid integrity to cause premature vRNA exposure and its subsequent degradation. In late stages of virus replication, high Cu^2+^ concentrations may interfere with the biogenesis of Zn^2+^-mediated viral BMCs required for nuclear transcription, export, translation, and trafficking and vRNA packaging by NC proteins. Indeed, many more viral proteins than those analyzed in Figure 1 possess juxtaposed PrLDs, ZnFs, and RRMs, and are either folded by or made functional by Zn^2+^ [46,165,167,168,169,170,171,172,173,174,175,176]. While acute increases in Cu/Zn ratios may resolve some viral infections, the model presents the herein described possibility that chronic unresolved infections may sustain high physiological Cu^2+^ concentrations. High Cu^2+^ that outcompetes Zn^2+^ for correct folding, activity, or multimerization of essential cellular proteins as BMCs may rather nucleate their aggregation as insoluble pathological plaques of neurodegenerative diseases (Figure 2A).

Indeed, virus infections have been shown to exacerbate neurodegenerative disease phenotypes [68,69,70,71,72], and just as Zn^2+^ supplementation has been successfully used as treatment against many viruses [160], Zn^2+^ supplementation is also described as a paradigm-shifting practice for neurodegenerative disease associated with Zn^2+^ and/or Cu^2+^ homeostasis abnormalities [316]. Elevated Cu/Zn ratios central to so many diseases, combined with the essential role of Zn^2+^ for BMC formation regulating many cellular processes, suggests that prolonged altered Cu/Zn ratios may represent an underappreciated and underlying feature of diseases (Figure 2). In addition to Cu-transporters highlighted in this report, many other Zn^2+^ metalloproteins and transporters are also modified by viral infections [269,317,318,319,320,321,322,323,324,325,326,327]. While research uncovering the basis for modified Cu/Zn ratios is important, it is possible that simple Zn^2+^ supplementation may assist in resolving its associated morbidities.

The model extends itself to propose countering viral transmission using environmental Cu^2+^. Cu^2+^ has historically been an easily procured effective antimicrobial and its anti-viral usage is supported by many demonstrations that this metal effectively inactivates all viruses tested. The current COVID-19 pandemic highlights an urgent need for methods reducing virus transmission. Mandatory face masks protect against virus aerosol droplets, but with evidence of SARS-CoV-2’s survival on certain surfaces for hours to days, and with copper surfaces showing the fastest inactivation [328], Cu^2+^ coating of reused face masks and shields may be more protective (Figure 2B). Finally, evidence suggests that the mechanism by which Cu^2+^ mediates virus inactivation is by its outcompeting virus-internalized structure-lending Zn^2+^, leading to premature virus uncoating and degradation of the consequently exposed vRNA (Figure 2C). Therefore, the application of Cu^2+^ coatings to touch surfaces may reduce transmission of current and future dangerous viruses while targeting drugs or vaccines become approved and available. Considering the global economic arrest caused by COVID-19, simpler strategies such as Zn^2+^ supplementation or Cu^2+^ surface coatings may keep future pandemics at bay.

## Supplementary Materials

The following are available online at https://www.mdpi.com/1999-4915/12/10/1179/s1, Table S1: List of most dangerous human viruses and their associated diseases, Table S2: Protein accession numbers for proteins and reference supporting ZnFs and RRMs.
