# Supplementary material for: Zinc and Copper Ions Differentially Regulate Prion-Like Phase Separation Dynamics of Pan-Virus Nucleocapsid Biomolecular Condensates
Source: Viruses. 2020 Oct 18;12(10):1179. doi: 10.3390/v12101179 (PMC7589941; doi:10.3390/v12101179)
Supplement: Supplementary file 1 [file viruses-12-01179-s001.pdf]

Supplementary Materials

Table S1

Table S1: List of most dangerous human viruses and their associated diseases			
**Species**	**Abbreviation**	**Family**	**Associated disease**
Influenza virus	Influenza	Orthomyxoviridae	seasonal flu (2019-2020) H1N1
Lassa mammarenavirus	LASV	Arenaviridae	Lassa hemorrhagic fever
Junín virus	JUNV	Arenaviridae	Argentine hemorrhagic fever
Machupo virus	MACV	Arenaviridae	Bolivian hemorrhagic fever
Hantaan orthohantavirus	HTNV	Hantaviridae	Korean hemorrhagic fever
Crimean-Congo hemorrhagic fever orthonairovirus	CCHFV	Nairoviridae	Crimean–Congo hemorrhagic fever
Varicella-zoster virus	VZV	Herpesviridae	varicella (shingles)
Human Papillomavirus 16	HPV16	Papillomaviridae	cervical cancer
Hepatitis B virus	HBV	Hepadnaviridae	hepatitis B
Variola virus	Smallpox	Poxviridae	Smallpox
Dengue virus	DENV	Flaviviridae	Dengue fever
West Nile virus	WNV	Flaviviridae	West Nile fever
Japanese encephalitis virus	JEV	Flaviviridae	Japanese encephalitis
Zika virus	ZIKV	Flaviviridae	Zika fever
Yellow fever virus	YFV	Flaviviridae	Yellow fever
Hepacivirus C	HCV	Flaviviridae	Hepatitis C
Rotavirus	RV	Reoviridae	Diarrhea
Ebola virus	EBOV	Filoviridae	Ebola virus disease
Marburg virus	MARV	Filoviridae	Marburg virus disease
Measles virus	MeV	Paramyxoviridae	Measles
Rabies lyssavirus	RABV	Rhabdoviridae	Rabies
Severe acute respiratory syndrome coronavirus-1	SARS-CoV-1	Coronaviridae	Severe acute respiratory syndrome
Severe acute respiratory syndrome coronavirus-2	SARS-CoV-2	Coronaviridae	COVID-19
MERS-coronavirus	MERS-CoV	Coronaviridae	Middle East respiratory syndrome
Human immunodeficiency virus -type 1	HIV-1	Retroviridae	Acquired immunodeficiency syndrome
Poliovirus	PV	Picornaviridae	Polio
Hepatovirus A	HAV	Picornaviridae	Hepatitis A

Table S2

Table S2: Protein accession numbers for proteins and reference supporting ZnFs and RRMs			
**Protein**	**Database**	**ID**	**References***
HIV-1 Gag	NCBI protein sequence database	GenBank: AD39400.1	[54]
SIV Gag	NCBI protein sequence database	GenBank: AEK79593.1	[54]
FIV Gag	NCBI protein sequence database	GenBank: AA48157.1	[54]
EIAV Gag	NCBI protein sequence database	GenBank: ACT31322.1	[54]
HTLV-1 Gag	NCBI protein sequence database	GenBank: AAB20767.1	[54]
RSV Gag	NCBI protein sequence database	GenBank: AA48534.1	[54]
MuLV Gag	NCBI protein sequence database	RefSeq: NP_040332.	[54]
SFVcpz Gag	NCBI protein sequence database	GenBank: AAA19977.1	[54,118]
BFV Gag	NCBI protein sequence database	GenBank: AWK77106.1	[54,118]
FFV Gag	NCBI protein sequence database	GenBank: GC11912.1	[54,118]
HPV16 L2	NCBI protein sequence database	GenBank: YP_009163895.1	[100,224]
HPV16 E6	NCBI protein sequence database	GenBank: ACQ90217.1	[305,306]
HPV16 E7	NCBI protein sequence database	GenBank: AAD33253.1	[305]
HBV core	NCBI protein sequence database	GenBank: CAA84790.1	[97]
DENV capsid	NCBI protein sequence database	GenBank: AGN94912.1	NR
WNV capsid	NCBI protein sequence database	GenBank: AMP46541.1	[103]
DENV NS3	NCBI protein sequence database	GenBank: ABV03585.1	NR
JEV NS3	NCBI protein sequence database	UniProt: Q66472	NR
HCV core	NCBI protein sequence database	GenBank: AFS60350.1	[102,103]
HCV NS5A	NCBI protein sequence database	GenBank: ABH10010.1	[78,90]
SARS-CoV-1 N	NCBI protein sequence database	GenBank: AYV99827.1	[224,307,308]
SARS-CoV-2 N	NCBI protein sequence database	GenBank: YP_009724397.2	NR
MERS N	NCBI protein sequence database	GenBank: QDI73608.1	NR

NoV VP1	NCBI protein sequence database	GenBank: AII73783.1	[223]
RVA VP6 core	NCBI protein sequence database	GenBank: AYA60744.1	[94]
RVA VP2 core	NCBI protein sequence database	GenBank: AYA60741.1	[92,309]
RVA NSP1	NCBI protein sequence database	GenBank: QDM35346.1	[77,93]
HTNV N	NCBI protein sequence database	GenBank: AAT37500.1	[310]
HTNV G1	NCBI protein sequence database	GenBank: CAA38922.1	[101]
Influenza A N	NCBI protein sequence database	GenBank: CAA91086.1	[96]
Influenza A M1	NCBI protein sequence database	GenBank: ABD59883.1	[79,82]
RABV N	NCBI protein sequence database	GenBank: QDF46327.1	NR
RABV P	NCBI protein sequence database	GenBank: QDF46328.1	NR
MeV V	NCBI protein sequence database	GenBank: AMM63617.1	NR
MeV N	NCBI protein sequence database	GenBank: AIG94078.1	[311,312]
MeV P	NCBI protein sequence database	GenBank: BAB60859.1	NR
MARV N	NCBI protein sequence database	GenBank: ABA87124.1	[105]
MARV VP30	NCBI protein sequence database	GenBank: CAA78118.1	NR
EBOV N	NCBI protein sequence database	GenBank: AAC09342.1	[99]
EBOV VP30	NCBI protein sequence database	GenBank: SCD11537.1	[105]

*References are present in cases when the RRM or ZnF is not identified from the NCBI protein sequence database. NR indicates reference not required as a result of ZnFs and RRMs positions indicated by NCBI protein sequence database
